# Serious Games Without Screens. Comment on “Involvement of End Users in the Development of Serious Games for Health Care Professions Education: Systematic Descriptive Review”

**DOI:** 10.2196/34656

**Published:** 2022-02-09

**Authors:** Michael Joseph Cosimini, Bjorn Watsjold, Teresa M Chan

**Affiliations:** 1 Oregon Health Sciences University Portland, OR United States; 2 Department of Emergency Medicine University of Washington Seattle, WA United States; 3 Division of Emergency Medicine Department of Medicine McMaster University Hamilton, ON Canada

**Keywords:** game-based learning, health professions education, participatory design, systematic review, user-centered design, serious games, game development, end users, education

Maheu-Cadotte et al [1] reviewed end user involvement in the development of serious games for health professionals’ education. They identified 45 games that were evaluated in randomized controlled trials for efficacy, of which only 21 reported on end user involvement during development. Citing a 2012 review, the authors included serious games defined as “video games designed with a primary educational purpose” [2]. This definition misses a large category of games whose review could provide important insights on the topic.

The term “serious games” likely originated from the 1970 monograph of the same name by Clark Abt [3]. The book concerns “*serious games* in the sense that these games have an explicit and carefully thought-out educational purpose and are not intended to be played primarily for amusement” [3]. Abt explored a number of examples including a card game to learn math, a board game to teach color mixing using transparent sheets, as well as a number of simulations of historic events and economies. He discussed a case with a component of computer-based simulation; however, the bulk of the book was dedicated to analog experiences. 

We would like to challenge the persistent usage of the narrow digital-only definition of a serious game seen in health professionals’ education, often citing the same 2012 review or its source, a 2006 book about the design of serious video games [4]. While the focus of *JMIR Serious Games* may be digital games and gamification, not all games are digital, and similarly, not all serious games are digital. As designers of card and board games used for health professionals’ education, we invite those in our field to reconsider this narrow definition. With its origin in mind, a more inclusive definition of serious games would be any standalone game designed with a specific purpose beyond entertainment *regardless of medium. *

We applaud Maheu-Cadotte et al [1] for highlighting the importance of end users’ contributions to game development, which applies to analog games as much as digital ones, with similar questions regarding balancing cost and possible benefit for consideration. Our experience has been variable: students (the target audience) co-designed *GridlockED* along with faculty but were only involved in playtesting during early design work on *Empiric* [5]. For *Clinical Coaching Cards,* the game underwent iterative rule changes based on observations of end user play and feedback collected during workshop sessions [6]. As Maheu-Cadotte et al [1] have shown, this is generally not well-reported, and we agree that end user involvement and feedback is a key component of demonstrating the validity of a game as an educational intervention.

We are appreciative of the insight provided by this review and hope the journal can prompt authors of future submissions to provide information pertaining to end user involvement on reports of serious games. We encourage other researchers to expand their definition of serious games and specify the focus on digital games when appropriate. 
